# Expression stability of putative reference genes in equine endometrial, testicular, and conceptus tissues

**DOI:** 10.1186/1756-0500-4-120

**Published:** 2011-04-12

**Authors:** Claudia Klein, Josep Rutllant, Mats HT Troedsson

**Affiliations:** 1 University of Kentucky, Department of Veterinary Science, 108 Gluck Equine Research Center, Lexington, KY, 40546, USA; 2 College of Veterinary Medicine, Western University of Health Sciences, 309 East Second Street, Pomona, CA 91766, USA

## Abstract

**Background:**

Quantitative RT-PCR data are commonly normalized using a reference gene. A reference gene is a transcript which expression does not differ in the tissue of interest independent of the experimental condition. The objective of this study was to evaluate the stability of mRNA expression levels of putative reference genes in three different types of equine tissue, endometrial, testicular, and conceptus tissue.

**Findings:**

The expression stability of four (uterine tissue) and six (testicular and conceptus tissue) was assessed using descriptive data analysis and the software programs Normfinder and geNorm. In uterine samples, *18S *showed the largest degree of variation in expression while *GAPDH*, *B2M*, and *ACTB *were stably expressed. *B2M *and *GAPDH *were identified as the most stably expressed genes in testicular samples, while *18S *showed some extent of regulation between samples. Conceptus tissue overall was characterized by very low variability of the transcripts analyzed with *GAPDH*, *YWHZ*, and 18S being the most stably expressed genes.

**Conclusions:**

In equine endometrium, *GAPDH*, *B2M*, and *ACTB *transcript levels are equally stable, while *18S *is less stably expressed. In testes and associated structures, *B2M *and *GAPDH *are the transcripts showing the least amount of variation, while in conceptus tissue *GAPDH*, *YWHZ*, and *18S *were identified as the most suitable reference genes. Overall, transcripts analyzed in conceptus tissue were characterized by less variation than transcripts analyzed in uterine and testicular tissue.

## Background

Real-time reverse transcription PCR (Real-time RT-PCR), also referred to as quantitative PCR (RT-qPCR) is a powerful tool to determine quantitative changes in mRNA expression levels and is widely applied in reproductive biology research. This technology provides a means to compare the abundance of a certain transcript of interest in tissue obtained from different biological statuses or to assess the impact of an experimental treatment on the expression of the gene of interest. Normalization procedures are an essential step in the analysis of real-time RT-PCR data to ensure that observed changes in transcript abundance reflect biological variation rather than systematic variation. Sources for systematic or non-biological variation include the amount of RNA input into the reverse transcription reaction, the efficiency of the reverse transcription reaction, pipetting accuracy while preparing the PCR reaction, and the presence of PCR inhibitory substances in a sample. Normalization procedures are therefore applied to correct for the occurrence of these systematic variations. A common normalization procedure is to report the expression level of the transcript of interest relative to the expression level of a reference gene. A reference gene, also referred to as maintenance or internal control gene, is a transcript whose expression level is constant in the specific tissue of interest, i.e. its mRNA abundance does not change with experimental treatment or with different biological conditions [1]. Stability of the reference gene/genes used in real-time RT-PCR events is crucial to avoid changes in gene expression of the transcript of interest due to their variation being interpreted as biological variation. Studies to determine reliable reference genes for equine skin and equine lymphocytes during exercise induced stress have been carried out [2,3]. There is no consensus regarding the most appropriate gene to use as an internal reference gene in RT-qPCR utilizing equine endometrial, testicular, or conceptus tissue samples. The objective of this study was to evaluate the stability of mRNA expression levels of putative reference genes in three different types of equine tissue, endometrial, testicular, and conceptus tissue.

## Materials and methods

### Animals and Tissue Collection

#### Collection of endometrium and conceptuses

All animal procedures were completed in accordance with and with the approval of the Institutional Animal Care and Use Committee at the University of Kentucky (protocol number 2008-0351). Endometrial tissue samples were recovered from cyclic mares 8 days (n = 4), 10 days (n = 5), 12 days (n = 6), 14 days (n = 5), and 16 days (n = 4) after ovulation, from pregnant mares endometrial tissue samples were recovered at Day 10 (n = 4), Day 12 (n = 4), and Day 16 (n = 3) of pregnancy. Pregnancy status was confirmed via transrectal ultrasonography. If no embryo was detected the mare was excluded from sample collection. Endometrial tissue samples were recovered transcervically using a punch biopsy instrument. Tissue specimens were snap-frozen in liquid nitrogen and stored at -80°C till further processing. Tissue samples were collected from a total of 17 different mares. Ten mares were used more than once for sample collection such that a total of 35 samples could be collected. Conceptuses were retrieved from pregnant mares through transcervical flush. Four conceptuses each were recovered 8, 10, 12, and 14 after ovulation.

#### Collection of testis and associated structures

Testes and associated structures were collected from mature stallions during routine castration and transported on ice to the lab within 30 min. Samples were obtained from testicular parenchyma (n = 11), head (n = 4), body (n = 4), and tail of the epididymis (n = 5), and the ductus deferens (n = 6). Tissue specimens were snap-frozen in liquid nitrogen and stored at -80°C till further processing.

### Isolation of RNA

Total cellular RNA from endometrial and testicular samples was isolated using Trizol reagent (Invitrogen, Carlsbad, CA) according to the manufacturer's recommendation followed by precipitation using an equal volume of isopropanol and 1/10 volume of 3 M sodium acetate. Total cellular RNA from conceptus tissue was isolated using RNeasy Mini kit (Quiagen, Valencia, CA). RNA was quantified via spectrophotometry using a NanoDrop ND-1000. Samples with a 260/280 ratio of 1.95 or greater and a 260/230 ratio of 2.0 or greater were used for analysis.

### Real-time RT-PCR

Real-time RT-PCR experiments were carried out under the consideration of the MIQE guidelines [4]. RNA samples (2 μg/reaction for endometrial and testicular specimens, and 200 ng/reaction for conceptus specimens) were treated with RNase-free DNase I (Ambion, Austin, TX) for 15 min at 37°C, heat denatured (75°C for 10 min), then reverse transcribed using High Capacity cDNA Reverse Transcription Kit and random hexamers (Applied Biosystems, Foster City, CA). cDNA was purified using the QIAquick^® ^PCR Purification Kit (Qiagen, Germantown, MD) and cDNA concentration was determined via spectrophotometry. Purified cDNA (50 ng) was used for each PCR reaction.

For endometrial specimen, the mRNA expression of four putative reference genes, glyceraldehyde 3-phosphate dehydrogenase (*GAPDHP*), 18S rRNA (*18S*), beta-2-microglobulin (*B2M*), and beta actin (*ACTB*) and one non-reference gene, solute carrier family 36 member 2 (*SLC36A2*) were measured by real-time RT-PCR. For testicular samples, the mRNA expression of *GAPDH*, *18S*, *B2M*, *ACTB*, Succinate dehydrogenase complex (*SDHA*), and beta glucoronidase (*GUSB*) as putative internal control genes and aromatase (*Cyp19a1*) as non-reference transcript were determined. For conceptus tissue, the expression of *GAPDH*, *18S*, *B2M*, *ACTB*, *SDHA*, and tyrosine 3-monooxygenase/tryptophan 5-monooxygenase activation protein (*YWHAZ*) as reference gene candidates and *Cyp19a1*as non-reference genes was assessed using quantitative PCR. Primers specific for the selected transcripts were designed using Jellyfish 3.3.1 (Field Scientific LLC, Lewisburg, PA) and are listed in Table 1. Specificity of the primers was confirmed via sequencing of the PCR products to confirm amplification of the intended target sequence. Primer efficiency was assessed using Linreg http://www.gene-quantification.de to ensure all primers resulted in PCR efficiencies of at least 1.9. Real-time PCR was completed using SYBR Green PCR Master Mix (Applied Biosystems) with the following cycling conditions: 95°C for 10 min; 40 cycles of 95°C for 15 sec, 59°C for 1 min; 55 to 95°C for dissociation. Each PCR was performed in triplicate. Specificity of amplification was monitored by including non-reverse transcribed RNA reactions for each sample and by completing a dissociation analysis at the end of each real-time run to verify the amplification of a single product. Cycle threshold (Ct) values were obtained through the auto Ct function.

**Table 1:** Primer sequences used in the current study.

Gene	GenBank accession #	Forward primer (5' to 3')	Reverse primer (3' to 5')	product size
*GUSB*	XM_001493514	GGGATTCGCACTGTGGCTGTCA	CCAGTCAAAGCCCTTCCCTCGGA	116

*Cyp19a1*	NM_001081805	GAGATGCCGTGGGAATTCTA	CCACGTTTCTCAGCCAAAAT	111

*ACTB*	NM_001081838	CGACATCCGTAAGGACCTGT	CAGGGCTGTGATCTCCTTCT	99

*B2M*	NM_001082502	GTGTTCCGAAGGTTCAGGTT	ATTTCAATCTCAGGCGGATG	102

*SLC36A2*	XM_001501324	GCTTCTGCCACAGGCTTAAC	CCGGCTTTGAGTCCATACAT	69

*GAPDH*	NM_001163856	AGAAGGAGAAAGGCCCTCAG	GGAAACTGTGGAGGTCAGGA	87

*SDHA*	XM_001490889	GCAGAAGAAGCCATTTGAGG	CCTGTCGATTACGGGTCTGT	103

*YHWAZ*	XM_001492988	TGTTGTAGGAGCCCGTAGGT	ATTCTCGAGCCATCTGCTGT	95

*18S*	XM_001497064	AACGACACTCTGGCATGCTAACTA	CGCCACTTGTCCCTCTAAGAA	98

### Data analysis

Data were analyzed separately but in the same fashion for endometrial, testicular, and conceptus tissue. Ct values from the ABI PRISM 7000 Sequence Detection System (Applied Biosystems) were exported into Excel (Microsoft Corporation, Redmond, WA). Excel and GraphPadPrism (La Jolla, CA) were used for further analysis of the dataset. Linreg was used to determine the efficiencies of each PCR reaction. Efficiency corrected Ct values from triplicate reactions were averaged for further analysis and are from now on referred to as Ct value. Mean Ct values, their standard error of measurement (SEM) and their coefficient of variation across the 35 samples were calculated for each of the putative reference genes. Ct values were logarithmically transformed and mean log transformed Ct values for each gene visualized using box and whisker plots. For each sample, ratios of mean Ct values among the putative reference genes were formed including all possible combinations. The mean and the coefficient of variation (CV) of the resulting ratios were calculated. Box and whisker plots were used to visualize the ratios of mean Ct values.

Two freely available software packages designed to identify stably expressed genes among a set of candidate genes, NormFinder http://www.mdl.dk/publicationsnormfinder.htm and geNorm http://medgen.ugent.be/~jvdesomp/genorm/, were used for further expression stability analysis. Bestkeeper [5] is another freely available software that determines stable reference genes through pair-wise correlation analysis. This software was not used in the current study to limit the extent of the manuscript.

#### NormFinder

NormFinder is publicly available software that uses an algorithm to identify the optimal normalization gene among a set of candidates. It ranks the set of candidate normalization genes according to their expression stability in a given sample set. The output provided by NormFinder includes an arbitrary stability value for each gene. The model and statistical framework underlying this software are described in Andersen et al. [6].

#### geNorm

geNorm is publicly available software package that determines the most stable genes from a set of tested genes. This application differs from NormFinder in that it determines the stability of the best pair of reference genes and not the most stable individual gene. The underlying principles and calculations are described in Vandesompele et al. [7].

#### Calculation of delta values

Delta Ct (ΔCt) values for *SLC36A2 *(endometrial samples), and *Cyp19a1 *(testicular and conceptus tissue) were calculated using each of the putative reference genes. Delta delta Ct (ΔΔCt) values relative to expression levels at Day 8 of the cycle (endometrial samples), ductus deferens (testicular samples), and Day 8 conceptus (conceptus tissue) respectively were then determined. Pearson's correlation coefficients were calculated for the resulting ΔΔCt values using SAS (SAS, Cory, NY).

## Results

### Endometrial samples

*18S *showed the highest level of mRNA abundance with a mean Ct value of 9.01 ± 0.25, whereas *GAPDH*, *ACTB*, and *B2M *mRNA showed lower expression levels with mean Ct values of 23.47 ± .21, 21.29 ± 0.23, and 20.64 ± 0.24, respectively.

**Figure 1:**
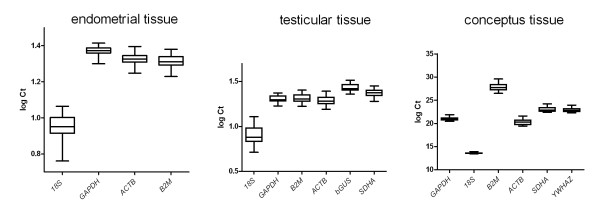
**Log transformed cycle threshold values for transcript evaluated among endometrial, testicular, and conceptus tissue samples**.

Mean Ct values for *18S *displayed the greatest amount of deviation with a CV of 17.07%. Mean Ct values for *GAPDH *displayed the least amount of variation with a CV of 5.24%. Mean Ct values for *ACTB *and *B2M *showed a similar degree of variability as those for *GAPDH*, with coefficient of variations of 6.39% and 7.04% (Table 2). Figure 1 displays the log transformed mean cycle threshold values for each of the four putative reference genes in the form of box and whiskers plots. Ct values are shown as median (lines), whereby the length of the box represents the 25^th ^and 75^th ^percentile, respectively. The symbol in the box indicates the mean. The vertical lines, i.e. whiskers, extend to the group minimum and maximum values. Upon subjective evaluation, *18S *showed the greatest degree of variation.

**Table 2:** Coefficient of variation of Ct values for transcripts tested in uterine, testicular, and conceptus tissue.

Uterine tissue		Testicular tissue		Conceptus tissue	
**Transcript**	**CV (%)**	**Transcript**	**CV (%)**	**Transcript**	**CV (%)**

*GAPDH*	5.24	*GAPDH*	8.72	*18S*	0.86
*ACTB*	6.39	*SDHA*	9.76	*GAPDH*	1.75
*B2M*	7.04	*GUSB*	9.89	*YWHZ*	1.87
*18S*	17.07	*B2M*	10.67	*SDHA*	2.23
		*ACTB*	12.37	*ACTB*	2.88
		*18S*	26.26	*B2M*	3.38

**Figure 2:**
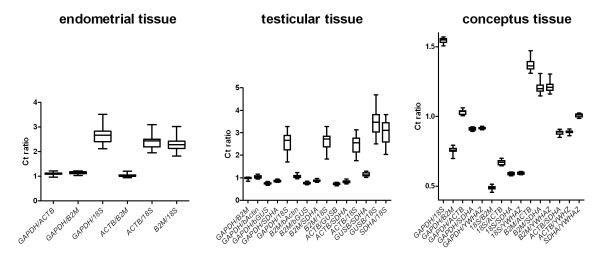
**Ratios of mean cycle threshold values for each possible combination of transcripts evaluated in endometrial, testicular and conceptus tissue**.

To evaluate expression stability, ratios of Ct values for all possible gene combinations were formed and graphed in the form of box and whiskers plots (Figure 2). The gene comparisons involving *18S rRNA *showed the greatest extent of variability. This observation was also reflected in an increased CV for the Ct ratios when *18S *was compared with the other genes. Mean Ct ratios including *18S *had an average CV of 13.29, whereas those ratios not including *18S *had an average CV of 4.37.

The stability values provided by NormFinder are listed in Table 3. *B2M *had the lowest stability value with 0.02, thereby being the most stable gene among the tested transcripts. *GAPDH *and *ACTB *had the same stability value of 0.028, which was slightly higher than the value for *B2M*. *18S *was ranked the least stable gene with the highest stability value of 0.06.

**Table 3:** Gene stability values returned by Normfinder.

Uterine tissue		Testicular tissue		Conceptus tissue	
**Transcript**	**Stability** **value**	**Transcript**	**Stability** **value**	**Transcript**	**Stability** **value**

*B2M*	0.020	*B2M*	0.018	*YWHZ*	0.003
*GAPDH*	0.028	*ACTB*	0.033	*GAPDH*	0.007
*ACTB*	0.028	*GAPDH*	0.039	*SDHA*	0.010
*18S*	0.060	*SDHA*	0.043	*18S*	0.013
		*GUSB*	0.043	*ACTB*	0.015
		*18S*	0.163	*B2M*	0.029

The lower the stability value, the more stable the gene is expressed.

**Figure 3:**
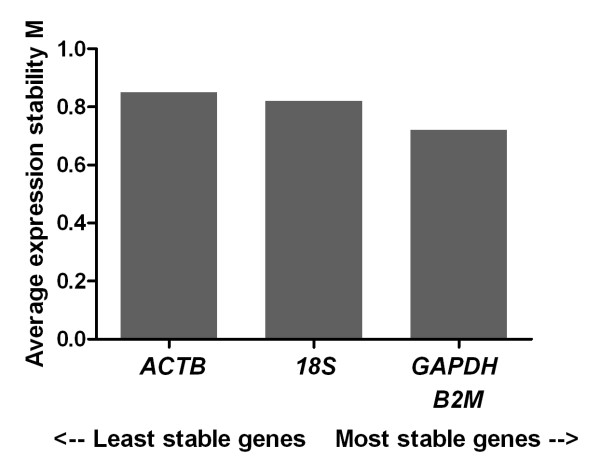
**Expression stability values (M) of the putative reference genes tested in uterine tissue**. Transcripts are ranked from the least stable to the most stable (left to right).

Upon analysis using geNorm, all four tested genes returned an average expression stability M value of less than 1.5, indicating stable expression across samples (Figure 3). geNorm determined *GAPDH *and *B2M *to be the most stably expressed pair of genes.

**Figure 4:**
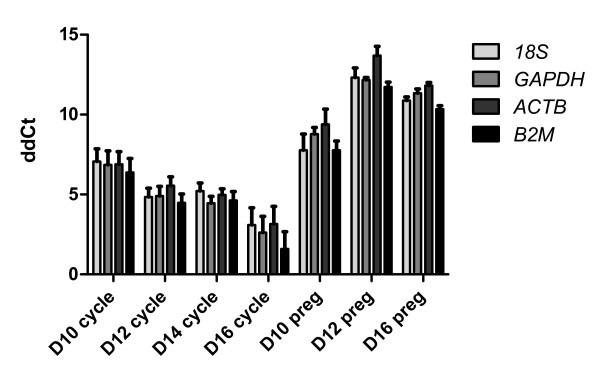
**Relative quantification of *SLC36A2 *expression in uterine tissue**. Displayed are average ΔΔCt values with SEM. ΔΔCt values generated using *GAPDH *for normalization showed the least amount of variability, with an average SEM of 1.06, while ΔΔCt values generated using *18S *for normalization showed the greatest degree of variability with an average SEM of 1.4.

To validate the above listed analysis concerning the stable expression of *18S*, *GAPDH*, *ACTB *and *B2M*, Ct values for *SLC36A2 *were determined and normalized using each of the four putative reference genes. Normalization was carried out by determining ΔCt values for *SLC36A2*. Expression levels of *SLC36A2 *were then determined relative to levels observed at Day 8 of the estrous cycle by generating ΔΔCt values. Pearson's correlation analysis of these ΔΔCt values revealed a high degree of correlation. Each of the six possible pair wise correlations showed an r-value of 0.99 and a p-value of less than 0.0001. Average ΔΔCt values for each of the four putative reference genes and seven types of endometrial tissue are shown in Figure 4.

### Testis and associated structures

*18S *showed the highest level of mRNA abundance with a mean Ct value of 8.19 ± 0.39, while the remaining transcripts showed similar abundance with a mean Ct value of 20.22 ± 0.32 (*GAPDH*), 20.67 ± 0.4 (*B2M*), 19.55 ± 0.44 (*ACTB*), 27.13 ± 0.49 (*GUSB*), and 23.65 ± 0.42 (*SDHA*) respectively.

Mean Ct values for *18S *displayed the greatest amount of deviation with a CV of 26.26%. Mean Ct values for *GAPDH *displayed the least amount of variation with a CV of 8.72%. The remaining transcripts showed a slightly higher CV than *GAPDH *(Table 2). Upon subjective evaluation of box and whiskers plots displaying the log transformed Ct values, *18S *showed the greatest degree of variation (Figure 1). Figure 2 shows box and whisker plots visualizing the mean ratios of all Ct values for each gene combination. Upon subjective evaluation the gene comparison involving *18S rRNA *showed the greatest extent of variability, which was reflected in an increased CV for mean Ct ratios including *18S *(15.75% versus 5.61% for Ct ratios not including *18S*).

The stability values returned by NormFinder (Table 3) identified *B2M *as the most stably expressed gene (0.018). *ACTB *and *GAPDH *had higher, i.e. less stable stability values than *B2M *(0.033 and 0.039), followed by *SDHA *and *GUSB *with a stability value of 0.043 each. *18S *was ranked the least stable transcript with a stability value of 0.163.

**Figure 5:**
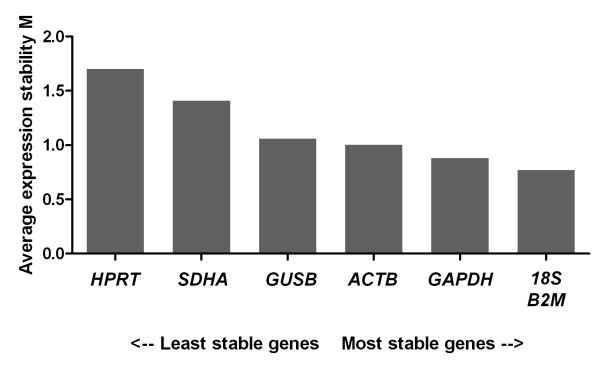
**Expression stability values (M) of the putative reference genes tested in testicular, epididymal, and ductus deferens tissue**. Transcripts are ranked from the least stable to the most stable (left to right).

Upon geNorm analysis, all six transcripts resulted in a M value of less than 1.5, equating stable expression (Figure 5). geNorm furthermore determined *B2M *and *18S *as the most stable gene combination.

**Figure 6:**
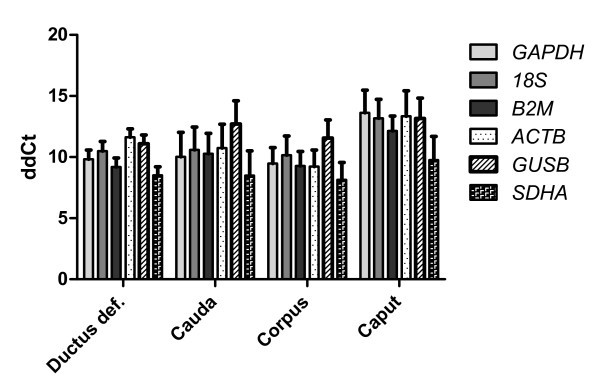
**Relative quantification of *Cyp19a1 *expression in testicular, epididymal, and ductus deferens tissue**. Displayed are average ΔΔCt values with SEM. ΔΔCt values generated using *B2M *for normalization showed the least amount of variability, with an average SEM of 1.2, while ΔΔCt values generated using *SDHA *and *ACTB *for normalization showed the greatest degree of variability with an average SEM of 1.52.

To validate the above listed analyses concerning the stable expression of *18S*, *GAPDH*, *ACTB*, *B2M*, *SDHA*, and *GUSB*, Ct values for *Cyp19a1 *were determined and normalized using each of the six putative reference genes. Normalization was carried out by determining ΔCt values for *Cyp19a1*. Expression levels of *Cyp19a1 *were then determined relative to levels observed in testis by generating ΔΔCt values. Pearson's correlation analysis of these ΔΔCt values revealed a high degree of correlation. Each of the fourteen possible pair wise correlations showed an r-value of 0.98 or higher and a p-value of less than 0.0001. Average ΔΔCt values for each of the four putative reference genes and four types testicular tissue are shown in Figure 6.

### Conceptus tissue

*18S *showed the highest level of mRNA abundance with a mean Ct value of 13.58 ± 0.03, while *B2M *exhibited the lowest levels with a mean Ct value of 27.81 ± 0.24.The remaining transcripts showed average expression levels with 20.97 ± 0.09 (*GAPDH*), 20.34 ± 0.15 (*ACTB*), 22.90 ± 0.11 (*YWHZ*), and 23.06 ± 0.13 (*SDHA*).

Mean Ct values for *18S *displayed the least amount of deviation with a CV of 0.86%. Mean Ct values for *GAPDH *displayed the greatest amount of variation with a CV of 3.38%. Mean Ct values for remaining transcripts showed as similar degree variation (Table 2). Figure 1 displays the log transformed mean Ct values for each of the four putative reference genes in the form of box and whiskers plots. Upon subjective evaluation *18S *showed the least, and *B2M *the greatest degree of variation.

To evaluate expression stability, ratios of Ct values for all possible gene combinations were formed and graphed in the form of box and whiskers plots (Figure 2). The gene comparisons involving *B2M *showed the greatest extent of variability. This observation was also reflected in an increased CV for the Ct ratios when *B2M *was compared with the other genes. Mean Ct ratios including *B2M *had an average CV of 13.29%, whereas those ratios not including *B2M *had an average CV of 4.37%.

The stability values provided by NormFinder are listed in Table 3. *YHWZ *had the lowest stability value with 0.003, thereby being the most stable gene among the tested transcripts. *B2M *was ranked the least stable gene with the highest stability value of 0.06.

**Figure 7:**
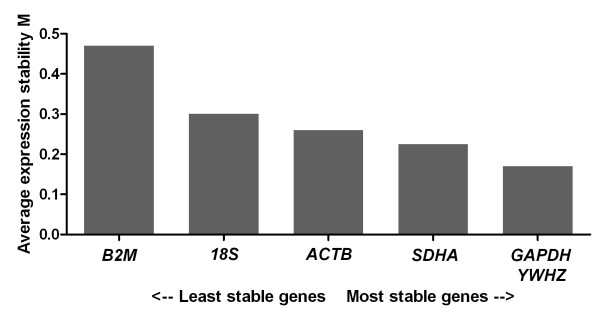
**Expression stability values (M) of the putative reference genes tested in conceptuses**. Transcripts are ranked from the least stable to the most stable (left to right).

Upon analysis using geNorm, all six tested genes returned an average expression stability M value of less than 1.5, indicating stable expression across samples (Figure 7). geNorm determined *GAPDH *and *YHWZ *to be the most stably expressed pair of genes, while *B2M *was ranked least stable.

**Figure 8:**
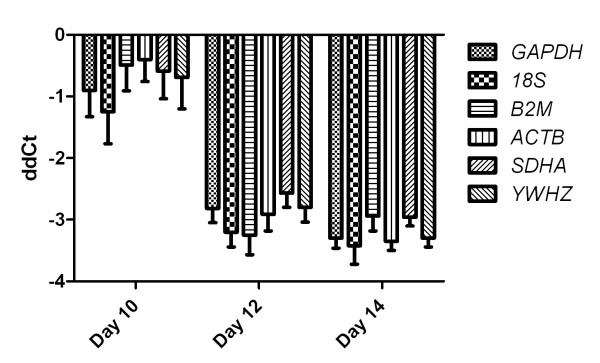
**Relative quantification of *Cyp19a1 *expression in conceptuses**. Displayed are average ΔΔCt values with SEM. ΔΔCt values generated showed similar degree of variability for all transcripts analyzed.

As described for uterine and testicular tissue, Ct values for *Cyp19a1 *were determined and normalized using each of the six putative reference genes. Normalization was carried out by determining ΔCt values for *Cyp19a1*. Expression levels of *Cyp19a1 *were then determined relative to levels observed in Day 8 conceptuses by generating ΔΔCt values. Pearson's correlation analysis of these ΔΔCt values revealed a high degree of correlation. Pair wise correlations no including B2M showed an r-value of 0.98 or greater and a p-value of less than 0.0001. Pair wise correlations including *B2M *showed an average r-value of 0.83 and a p-value of 0.001. Average ΔΔCt values for each of the six putative reference genes and three developmental stages of conceptus development are shown in Figure 8.

## Discussion

Real time RT-PCR is one of the most sensitive and reproducible quantification methods for gene expression analysis. Selection of internal control genes is a crucial step in the experimental design process, as relative quantification procedures rely on the stable expression of such internal control or reference genes among samples regardless of the biological conditions. The most suitable reference gene has to be determined for each tissue as a gene stably expressed in one type of tissue is not necessarily stably expressed in another type of tissue. However, control genes are commonly chosen on the basis of observations from research on other tissue types or on the same tissue type in a different species. The present study was performed to determine the suitability of selected putative internal control genes in three different types of tissues when performing real-time RT-PCR analysis in the equine species.

### Uterine samples

*18S rRNA*, *GAPDH*, *ACTB*, and *B2M *are commonly used as internal control genes when performing quantitative PCR analysis on uterine tissue samples derived from species such as human or mouse. As expected, *18S rRNA *showed the highest level of mRNA abundance while *GAPDH*, *ACTB*, and *B2M *showed lower expression levels. The average cycle threshold value across all 35 analyzed samples showed the greatest degree of variability for *18S rRNA *whereas the remaining three transcripts displayed a low degree of variation. Ct ratios for all possible pair wise combinations were formed. Assuming stable expression, one would expect a low variability in the resulting ratios. However, a pattern emerged, whereby comparisons including *18S *displayed increased deviation, and comparisons excluding *18S *showed low deviation. This is in accordance with the greater degree of variability observed for *18S *Ct values. NormFinder, a publicly available program that ranks transcripts according to their expression stability, returned *18S *as the least stably expressed gene supporting the results of the above mentioned descriptive analyses. *B2M *was listed the most stable expressed gene, closely followed by *GAPDH *and *ACTB*. geNorm identified *GAPDH *and *B2M *as the most stably expressed pair of genes. Overall, all four genes were identified as stable amongst the samples analyzed with *18S *being slightly less stably expressed than *GAPDH*, *B2M*, and *ACTB*.

To validate the suitability of the tested transcripts as reference genes, expression levels for *SLC36A2 *were determined. *SLC36A2 *had previously been identified as highly up-regulated during early pregnancy and was chosen with the hypothesis that its expression levels would vary throughout the estrous cycle and early pregnancy [8]. Cycle threshold values for *SLC36A2 *were normalized using each of the four putative reference genes through generating ΔCt values. Expression values relative to levels observed at Day 8 of the estrous cycle were determined through determining ΔΔCt values. Pair wise comparisons of resulting ΔΔCt values revealed that all six possible pair wise combinations were highly correlated. High correlation of ΔΔCt values reflects the similar expression of all putative reference genes across the tested samples. Average ΔΔCt values for each of the seven biological conditions showed the greatest degree of variation when *18S *was used for normalization, while *GAPDH *as normalization factor resulted in the least degree of variability. This finding supports the above stated conclusion that all four genes were identified as stably amongst the samples analyzed with *18S *being slightly less stably expressed than *GAPDH*, *B2M*, and *ACTB*.

Surprisingly few studies concerning the ideal selection of reference genes in endometrial tissue have been published. Murray and others [9] evaluated the appropriateness of several transcripts as reference genes on endometrial tissue specimens obtained from women throughout the menstrual cycle and then tested the suitability of these genes to detect the variation of estrogen and progesterone receptor levels during the course of the cycle. geNorm determined *GAPDH *to be amongst the most stably expressed genes, which is in agreement with the results of the current study in horse tissues. *ACTB*, which expression was ranked as stable according to geNorm, however failed to detect changes in endometrial estrogen and progesterone receptor levels and was therefore determined to be not suitable as a reference gene. This underlines the importance to not only test the stability of a putative reference gene for a specific tissue type, but also the necessity to evaluate each species individually. Walker and co-workers evaluated a set of endogenous control genes for bovine endometrial tissue. The transcripts tested in that study however did not overlap with those tested in the present study so that a comparison of results is not possible [10]. The expression of *18S*, the transcript showing the greatest variation across the 35 samples analyzed in this study, has been identified as regulated by progesterone in the murine uterus [11]. This observation may provide some explanation for the observed variation of *18S *in equine endometrial tissue samples collected throughout the estrous cycle, as these samples have been exposed to endogenous progesterone of luteal origin for variable time periods.

### Testis and associated structures samples

Gene expression levels of *18S*, *GAPDH*, *B2M*, *ACTB*, *GUSB*, and *SDHA *were measured in 30 tissue samples collected from testis, epididymis, and ductus deferens. As expected, *18S rRNA *showed the highest level of mRNA abundance. The average cycle threshold value across all samples analyzed showed the greatest degree of variability for *18S rRNA *whereas the remaining four transcripts displayed a low, and *GAPDH *the lowest degree of variation. Ct ratios for all possible pair wise combinations were formed, whereby comparisons including *18S *displayed increased deviation, and comparisons excluding *18S *showed low deviation. This is in accordance with the greater degree of variability observed for *18S *Ct values. NormFinder identified *18S *as the least stably expressed gene supporting the results of the above mentioned descriptive analyses. Upon geNorm analysis, *18S*, *B2M *and *GAPDH *were listed as the most stably expressed genes. Surprisingly, geNorm identified *B2M *and *18S *as the most stably expressed pair of genes. This finding disagrees with NormFinder, which ranked *18S *as the least stable transcript and in addition does not match the descriptive analysis of the dataset which identified *18S *as the gene with the largest degree of variability. This is the only case throughout this data analysis in which results returned by NormFinder and geNorm do not match. The descriptive analysis however supports the results returned by NormFinder.

As performed for the analysis on uterine tissue, the suitability of the tested transcripts as reference genes was validated through determining the relative expression levels for a non-reference transcript, in this case the relative expression of *Cyp19a1 *to levels observed in testis. *Cyp19a1*, an enzyme involved in the biosynthesis of estrogens was chosen because of its known high level of expression in testicular tissue. Pair wise comparisons of resulting ΔΔCt values revealed that all thirteen possible pair wise combinations were highly correlated.

The authors were not able to find information regarding the evaluation of internal control genes in testicular, epididymal, or ductus deferens tissue, neither in the horse nor in any other species. Overall, the current analysis revealed *B2M *and *GAPDH *as the most stable expressed transcripts in the set of tissue used and are therefore recommended to use as internal control genes.

### Conceptuses

As seen for uterine and testicular tissue, *18S *showed the highest mRNA abundance. *B2M *exhibited lowest mRNA levels with the remaining transcripts at intermediate levels. Unlike the other types of tissues tested *18S *mRNA showed the least amount of variation among the transcripts analyzed with a CV of mean Ct values of less than one percent. *B2M *showed the greatest degree of variability during the course of the descriptive analysis which became especially evident when forming ratios of Ct values for all possible gene combinations. Likewise, *B2M *was identified as the least stable expressed gene by NormFinder and geNorm, while *YWHZ *and *GAPDH *were determined to be the most stable transcripts. Despite the descriptive analysis, which revealed *18S *as the transcript with the least amount of variation, i.e. the most stable gene, both NormFinder and geNorm did not rank *18S *as the most stably expressed gene. *Cyp19a1 *mRNA levels were employed to determine relative expression levels for a non-reference transcript, as abundance for this transcript had previously been identified to change with conceptus development (Klein, unpublished observation). Formation of ΔΔCt values relative to Day 8 conceptuses for *Cyp19a1 *and their pair wise comparisons revealed high correlation for combinations not including *B2M *normalized ΔΔCt values, further corroborating the above listed findings.

Overall, the six reference genes tested in conceptus' tissue showed less variability in expression than the reference genes tested in uterine and testicular samples. Pre-implantation conceptuses are less complex in their composition than uterine and testicular tissues, most likely contributing to the decreased variability. Smits and co-workers [12] determined the expression of 8 candidate reference genes in equine blastocysts which is a stage of embryonic development equivalent to the Day 8 conceptuses in the current study. According to the M-value returned by geNorm, transcripts analyzed in that study were less stably expressed than in the current study. In the present study, cDNA was quantified after reverse transcription, thereby providing more control over the amount of cDNA used as input for each RT-qPCR reaction. The finding by Smits et al. that *SDHA *appeared to be highly regulated in equine blastocysts, could not be confirmed and is in line with the stable expression of *SDHA *described for bovine embryos [13].

In general the current analysis revealed *18S*, *GAPDH*, and *YWHZ *to be the most stable transcript in pre-implantation equine embryos and are recommended as reference genes for RT-qPCR experiments.

## Competing interests

None of the authors in this study has any financial personal relationship with any organization that could influence (bias) this work.

## Authors' contributions

CK designed and conducted the study. JR participated in design of the study and drafting the manuscript. MHT conceived the study, participated in its coordination and helped to draft the manuscript. All authors read and approved the final manuscript.

## References

[B1] Ferre F Quantitative or semi-quantitative PCR: reality versus myth PCR Methods Appl 1992 2 1 9 1490169 10.1101/gr.2.1.1

[B2] Bogaert L Van Poucke M De Baere C Peelman L Gasthuys F Martens A Selection of a set of reliable reference genes for quantitative real-time PCR in normal equine skin and in equine sarcoids BMC Biotechnol 2006 6 24 10.1186/1472-6750-6-24 16643647 PMC1484482

[B3] Cappelli K Felicetti M Capomaccio S Spinsanti G Silvestrelli M Supplizi AV Exercise induced stress in horses: selection of the most stable reference genes for quantitative RT-PCR normalization BMC Mol Biol 2008 9 49 10.1186/1471-2199-9-49 18489742 PMC2412902

[B4] Bustin SA Benes V Garson JA Hellemans J Huggett J Kubista M Mueller R Nolan T Pfaffl MW Shipley GL The MIQE guidelines: minimum information for publication of quantitative real-time PCR experiments Clin Chem 2009 55 611 622 10.1373/clinchem.2008.112797 19246619

[B5] Pfaffl MW Tichopad A Prgomet C Neuvians TP Determination of stable housekeeping genes, differentially regulated target genes and sample integrity: BestKeeper--Excel-based tool using pair-wise correlations Biotechnol Lett 2004 26 509 515 10.1023/B:BILE.0000019559.84305.47 15127793

[B6] Andersen CL Jensen JL Orntoft TF Normalization of real-time quantitative reverse transcription-PCR data: a model-based variance estimation approach to identify genes suited for normalization, applied to bladder and colon cancer data sets Cancer Res 2004 64 5245 5250 10.1158/0008-5472.CAN-04-0496 15289330

[B7] Vandesompele J De Preter K Pattyn F Poppe B Van Roy N De Paepe A Speleman F Accurate normalization of real-time quantitative RT-PCR data by geometric averaging of multiple internal control genes Genome Biol 2002 3 RESEARCH0034 10.1186/gb-2002-3-7-research0034 12184808 PMC126239

[B8] Klein C Scoggin KE Ealy AD Troedsson MH Transcriptional Profiling of Equine Endometrium During the Time of Maternal Recognition of Pregnancy Biol Reprod 2010 10.1095/biolreprod.109.081612 20335638

[B9] Murray RS Inman DA Yuan L Fritz MA Young SL Choice of a constitutive housekeeping gene (HKG) is critical in the analysis of real-time reverse transcriptase polymerase chain reaction (qRT-PCR) results Fertil Steril 2010 86 3 S38 S39 10.1016/j.fertnstert.2006.07.106

[B10] Walker CG Meier S Mitchell MD Roche JR Littlejohn M Evaluation of real-time PCR endogenous control genes for analysis of gene expression in bovine endometrium BMC Mol Biol 2009 10 100 10.1186/1471-2199-10-100 19878604 PMC2774697

[B11] Craythorn RG Girling JE Hedger MP Rogers PA Winnall WR An RNA spiking method demonstrates that 18S rRNA is regulated by progesterone in the mouse uterus Mol Hum Reprod 2009 15 757 761 10.1093/molehr/gap058 19602508

[B12] Smits K Goossens K Van Soom A Govaere J Hoogewijs M Vanhaesebrouck E Galli C Colleoni S Vandesompele J Peelman L Selection of reference genes for quantitative real-time PCR in equine in vivo and fresh and frozen-thawed in vitro blastocysts BMC Res Notes 2009 2 246 10.1186/1756-0500-2-246 20003356 PMC2797813

[B13] Goossens K Van Poucke M Van Soom A Vandesompele J Van Zeveren A Peelman LJ Selection of reference genes for quantitative real-time PCR in bovine preimplantation embryos BMC Dev Biol 2005 5 27 10.1186/1471-213X-5-27 16324220 PMC1315359

